# Quadrilateral Segmental Analysis (QSA): A Calibration-Free Geometric Method for Detecting Lumbar Spondylolisthesis

**DOI:** 10.3390/diagnostics16203291

**Published:** 2026-10-10

**Authors:** Tomasz Szczepański, Michał Jasiński, Marta Koźba-Gosztyła, Jakub Więcław, Piotr Szymczyk, Joanna Bladowska, Bogdan Czapiga

**Affiliations:** 1 Clinical Department of Neurosurgery, Center for Neurologic Disorders, 4th Military Clinical Hospital, 50-981 Wroclaw, Poland; martakozba1@o2.pl (M.K.-G.); wieclawjakub@gmail.com (J.W.); szymczyk23@gmail.com (P.S.); bogdan.czapiga@pwr.edu.pl (B.C.); 2 Department of Radiology and Imaging Diagnostics, 4th Military Clinical Hospital, 50-981 Wroclaw, Poland; jasinsmichal@gmail.com; 3 Department of Preclinical Sciences, Pharmacology and Medical Diagnostics, Faculty of Medicine, Wroclaw University of Science and Technology, 50-370 Wroclaw, Poland; joanna.bladowska@pwr.edu.pl; 4 Department of Neuro-Oncology Imaging, 4th Military Clinical Hospital, 50-981 Wroclaw, Poland; 5 Department of Radiology, T. Marciniak Lower Silesian Specialist Hospital, 54-049 Wroclaw, Poland; 6 Department of Clinical Neurosciences, Faculty of Medicine, Wroclaw University of Science and Technology, 50-370 Wroclaw, Poland

**Keywords:** quadrilateral segmental analysis (QSA), degenerative spondylolisthesis, anterolisthesis, segmental instability, standing radiography, diagnostic accuracy, quantitative imaging, lumbar spine

## Abstract

**Background/Objectives**: On standing lateral radiographs, millimetre measurements of vertebral slip depend on magnification and calibration; slip percentage is scale-free but is usually reported in coarse Meyerding grades. In this exploratory derivation study, we introduce quadrilateral segmental analysis (QSA), a calibration-free geometric method for detecting anterolisthesis. **Methods**: In 50 patients with degenerative spondylolisthesis from the PROGRES cohort, a blinded observer drew the L1–S1 superior endplate lines on standing radiographs; an automated pipeline computed the diagonal ratio K and the posterior angles of each quadrilateral formed by adjacent lines. The annotation was performed by a single observer; its reproducibility was not assessed, and its sensitivity to annotation error was simulated. The reference standard was an adjudicated two-radiologist classification (κ = 0.916). Segments outside the anterior-translation domain (negative shear; n = 90) were not graded further; the remaining 160 were analysed with patient-cluster bootstrap and leave-one-patient-out validation. **Results**: Anterolisthesis was present in 54 of 160 segments (33.8%). K discriminated well (area under the curve [AUC] 0.892, 95% CI 0.841–0.937): K ≤ 0.85 supported a slip (specificity 99.1%), and K > 0.95 argued against it (sensitivity 100%; smallest reference-positive slip 9%). Rounded to one decimal, K assigned 46.9% of segments to extreme tiers without observed misclassification. With the lower posterior angle N ≤ 93° in the intermediate tier, the two-step rule reached 94.4% sensitivity and 95.3% specificity, unchanged under nested leave-one-patient-out validation. Simulated endpoint errors of 0.5–1.0 mm changed the two-step classification in 5–13% of segments (ICC 0.74–0.92 for K and 0.85–0.96 for N). **Conclusions**: Calibration-free QSA parameters discriminated reference-positive from reference-negative segments in patients with degenerative spondylolisthesis. K (the diagonal ratio) translates the radiologist’s assessment of anterolisthesis into a single number. These exploratory findings, which do not establish performance for small or borderline slips, require replication in independent cohorts and by other observers before the thresholds can be applied clinically.

## 1. Introduction

Degenerative lumbar spondylolisthesis is common in adults over 50 years of age and is associated with low back pain and neurogenic claudication [1,2,3,4]. The choice between conservative care, decompression alone and decompression with fusion is guided in part by whether—and how confidently—the slip is detected [5,6,7,8].

In routine practice, however, the slip is still judged visually or against millimetre thresholds—typically ≥ 3 mm [4,9]—whose validity depends on image scaling: lumbar radiographic magnification varies widely between patients and rises with body mass index [10]; measurements scaled with default Digital Imaging and Communications in Medicine (DICOM) factors deviate substantially from marker-calibrated values [11]; and inconsistent handling of magnification and landmarks contributes to the wide spread of published prevalence estimates [1].

Instability judgements built on these readings remain largely subjective and depend on the imaging protocol [12,13], and in secondary analyses of the NORDSTEN-DS trial, neither established instability criteria nor surgeons’ individual recommendations identified patients who benefited from additional fusion [14,15]. Objective classification requires reproducible metrics anchored in normative reference data [16]. Slip percentage is independent of scale, but it reduces each segment to a single ratio of translation to endplate depth and, when graded by Meyerding, to 25% steps [3].

**Figure 1:**
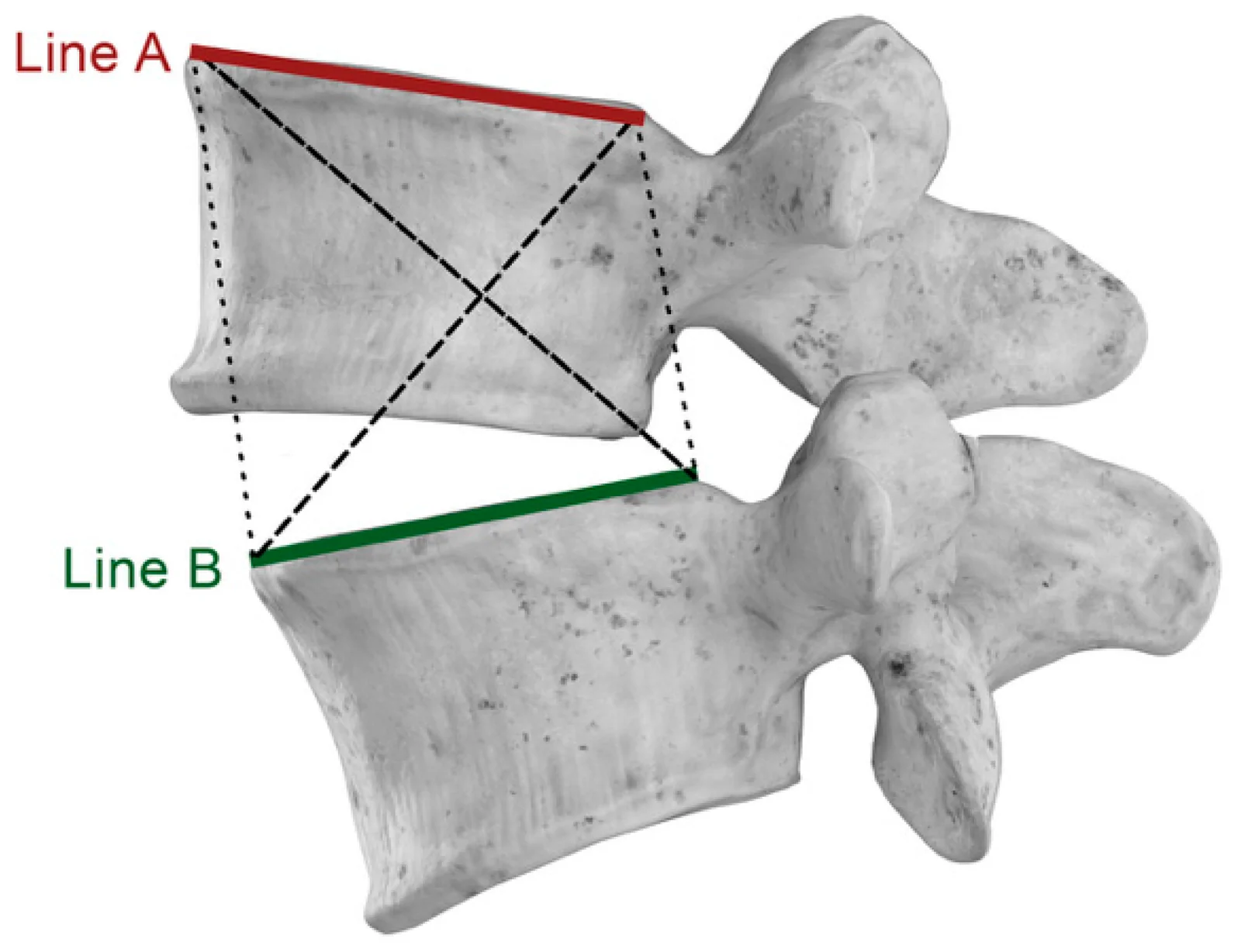
Principle of quadrilateral segmental analysis (QSA) on a lumbar motion segment. A straight line is drawn along the superior endplate of the cranial (line A) and caudal (line B) vertebrae; the lines joining their anterior and posterior endpoints (dotted) close the segmental quadrilateral, whose diagonals (dashed) define the diagonal ratio K.

We therefore propose quadrilateral segmental analysis (QSA): a single straight line is drawn along the superior endplate of each vertebra, and every motion segment is represented by the quadrilateral formed by two adjacent endplate lines and the connections of their anterior and posterior endpoints (Figure 1). Anterior translation skews this quadrilateral—the upper posterior interior angle M opens, the lower posterior angle N closes, and the diagonals become unequal (ratio K)—so translation is expressed by angles and dimensionless ratios that are inherently independent of calibration.

The aim of this exploratory derivation study was to define the QSA parameters, to estimate with internal validation how well they discriminate reference-positive from reference-negative segments in patients with degenerative lumbar spondylolisthesis and to derive candidate thresholds for external validation.

## 2. Materials and Methods

### 2.1. Design and Participants

This exploratory derivation study with internal validation used prospectively collected data from the PROGRES cohort (an evaluation of the natural course of lumbar degenerative spine disease and factors influencing its progression, with an analysis of the impact of surgical intervention as a modifying factor in the long-term course of the disease), an ongoing prospective observational cohort study conducted at the Neurosurgical Department of the 4th Military Clinical Hospital in Wroclaw, Poland. This study was approved by the Bioethics Committee of the Lower Silesian Medical Chamber in Wroclaw, Poland (approval no. 09/DOBD/2025, dated 9 April 2025) and conducted in accordance with the Declaration of Helsinki. Every human participant provided written consent prior to enrolment. The study protocol was developed in accordance with the Strengthening the Reporting of Observational Studies in Epidemiology (STROBE) guidelines for cohort studies (Supplementary Checklist S1).

The present analysis uses preoperative assessment data collected between May 2025 and May 2026. Fifty consecutive adult patients with degenerative lumbar spondylolisthesis were included. Exclusion criteria included isthmic spondylolisthesis, deformity, previous spinal surgery, spinal neoplasms and neurological or muscular diseases causing motor impairment (e.g., amyotrophic lateral sclerosis, multiple sclerosis and myopathies). Because all participants had degenerative spondylolisthesis, this study estimates discrimination between reference-positive and reference-negative segments within affected patients, not diagnostic performance in an unselected population assessed for suspected disease. The PROGRES cohort was used because it provided consecutive prospective enrolment, standing lateral radiographs acquired under one predefined protocol, predefined exclusion of conditions that distort segmental geometry and blinded double reading of every segment for the reference standard; its longitudinal follow-up will allow the same parameters to be related to progression, treatment and outcome. The analysis does not depend on features unique to this cohort and can be repeated in other cohorts of patients with degenerative spondylolisthesis. Demographic and radiographic characteristics of the cohort are summarized in Table 1.

### 2.2. Radiographs and Reference Standard

Each patient underwent standing lateral radiography of the lumbar spine in a standardized free-standing position. No distance calibration was required because every index parameter is an angle or a dimensionless ratio.

The reference standard was constructed as follows. Two experienced radiologists, blinded to the QSA output and to each other’s ratings, independently reviewed every radiograph and classified each of the five motion segments from L1–L2 to L5–S1 as showing anterolisthesis or not, in accordance with recommendations for the construction and reporting of the reference standard in diagnostic accuracy studies [18,19]. Both readers assessed each segment visually and holistically, guided by eight pre-specified features with written operational definitions (File S1). Three were geometric: anterior translation, with ≥3 mm or a slip of ≥5% as alternative thresholds [20], judged between the posterior corners of adjacent vertebrae according to Meyerding [21]; alignment of the posterior vertebral body line, tangential to the posterior borders of the vertebral bodies; and anterior–posterior disc height asymmetry [22]. Five were non-geometric: vacuum disc phenomenon [23,24], disc space narrowing [25], traction spur [26], spinous process step-off [27] and Baastrup sign [28,29]. The first two are direct signs of translation; the other six mark segmental degeneration and instability [30,31]. Each reader integrated the features into a binary verdict (anterolisthesis present or absent), without a fixed scoring rule and without digital measurement tools. Segments on which the two readers disagreed were referred to a third, senior radiologist, who reviewed the same radiograph while blinded to the index test and to the identity of the disagreeing readers, and whose verdict became final; adjudicated panel diagnosis is an accepted strategy when a single, error-free reference test is unavailable [32,33]. Because the thresholds were alternatives and 3 mm exceeds 5% of any lumbar endplate depth, the scale-free criterion was sufficient and required no calibration. Indirect findings informed the judgement but could not establish positivity without anterior translation; segments near the threshold were classified by holistic judgement, and discordant verdicts were adjudicated by the third radiologist. The adjudicated segment-level classification constituted the reference standard for all subsequent analyses.

### 2.3. Index Test: Quadrilateral Segmental Analysis

A single experienced spine surgeon, blinded to the radiologists’ ratings, drew one straight line along the superior endplate of L1, L2, L3, L4, L5 and S1—six lines per patient. Each line ran along the superior endplate from its anterior to its posterior rim, excluding osteophytes; on concave endplates, it joined the anterior and posterior rim points; double contours were resolved by drawing the line midway between them, and at S1 the line ran from the posterosuperior to the anterosuperior corner of the sacral endplate (File S1). Annotated images were processed by a custom automated Python pipeline that detected the lines by color segmentation and fitted them by principal component analysis. The endpoints of each line were the extreme projections of the annotated pixels on its principal axis.

**Figure 2:**
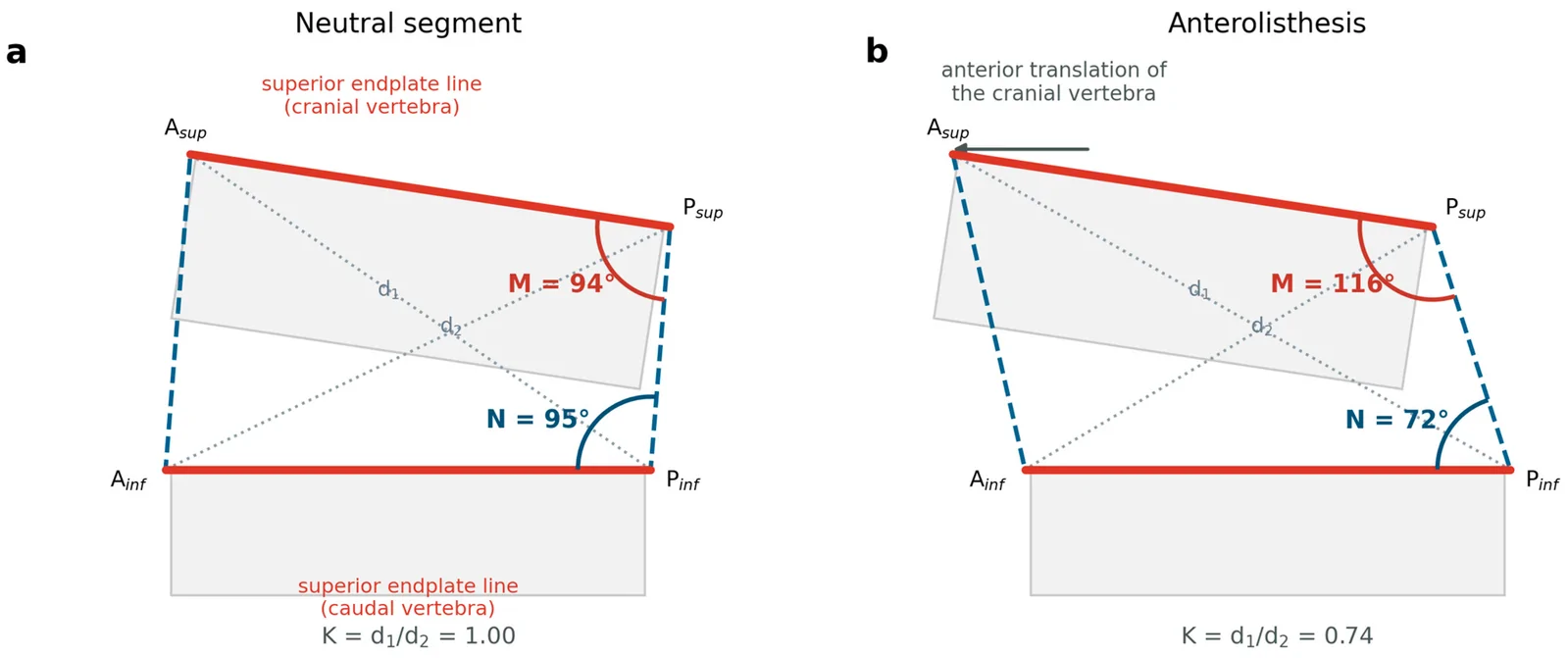
QSA construction. (**a**) Configuration with equal diagonals (K = d_1_/d_2_ = 1), in which M ≈ N; with equal endplate lengths the diagonals are equal only when M = N. A lordotic segment without translation has M = 90°, N = 90° + α and K < 1 (Appendix A). (**b**) Anterolisthesis: anterior translation of the cranial vertebra opens M, closes N and makes the diagonals unequal (K < 1). In both configurations M + N = 180° + segmental rotation.

For each motion segment, the quadrilateral A_sup_–P_sup_–P_inf_–A_inf_ was constructed from the anterior (A) and posterior (P) endpoints of the cranial and caudal endplate lines (Figure 2). The QSA parameters were: M, the interior angle at the upper posterior vertex; N, the interior angle at the lower posterior vertex; M − N, the posterior angular asymmetry; K, the ratio of the shorter to the longer diagonal (0 < K ≤ 1); and L, the angle between the diagonals at their intersection. The direction-aware ratio K_dir_ = d_1_/d_2_, with d_1_ = |A_inf_P_sup_| and d_2_ = |P_inf_A_sup_|, gives K = min(K_dir_, 1/K_dir_). K = 1 denotes equal diagonals, not absent translation: K also depends on segmental angle and endplate lengths (Appendix A). Conventional descriptors computed from the same two lines served as comparators: segmental rotation α (the dihedral angle between the endplate lines), total rotation, wedging; tilt; and the two translation measures: shift (F), a slip-percentage analogue expressed as a percentage of caudal endplate length; and shear (D), the displacement between the endplate midpoints expressed as a percentage of cranial endplate length, with anterior translation of the cranial vertebra defined as positive. For the comparison of predictors, translation measures were used as absolute values (|D|, |F|) and the upper posterior angle as its deviation from the neutral value, |M − 95°|. Equations and sign conventions are given in Appendix A.

The identity M + N = 180° + α holds by construction for every quadrilateral (Appendix A); it was used only as a computational check and does not verify landmark placement.

QSA rests on three geometric assumptions. First, like any measurement on a lateral radiograph, it captures only the sagittal component of displacement: coronal translation lies along the beam and is not visible, and axial rotation shortens the projected endplate and separates the left and right posterior corners. Second, each endplate is assumed to project as a single contour; with asymmetric degeneration or rotation the rims separate, and the line drawn midway between them represents the average of the two sides. Third, the superior and inferior endplates of a vertebral body need not be parallel because each quadrilateral is built from the superior endplates of two adjacent vertebrae and the wedge of the cranial vertebral body is contained in α; QSA assumes instead that the posterior wall of the cranial vertebra is approximately perpendicular to its superior endplate. A deviation δ shifts the neutral value of N by δ and changes F by about 1.4–1.8 percentage points per degree (Appendix A, Equation (A9)).

For cohort description only, the same pipeline additionally computed the global spinopelvic parameters from the endplate lines together with a single mark on the femoral heads: lumbar lordosis (LL, L1–S1) with its proximal (PLL, L1–L4) and distal (DLL, L4–S1) arcs, sacral slope (SS), pelvic tilt (PT), pelvic incidence (PI) and the lordosis distribution index (LDI = DLL/LL × 100) [17]. These parameters were not used in any diagnostic analysis; their internal consistency was verified through the identity PI = SS + PT.

### 2.4. Direction of Translation and Analysis Set

**Figure 3:**
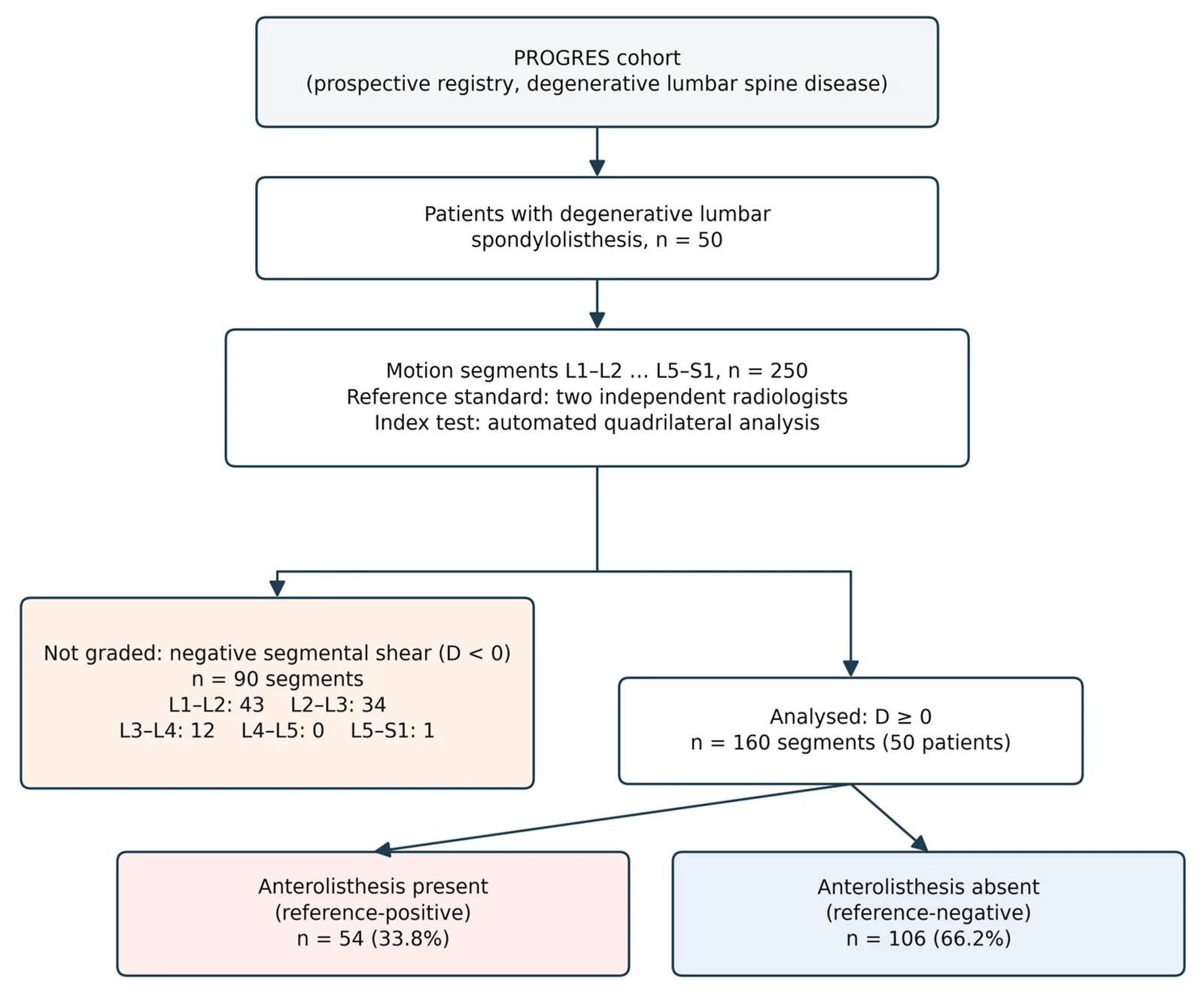
Flow of patients and motion segments (STARD diagram). Segments with negative shear (D < 0) were not graded further.

The target condition was anterior translation. Because K is direction-blind, the direction of translation was first established from the sign of the shear D, computed from the same two endplate lines without reference information. Segments with D < 0 (n = 90 of 250; Figure 3) were not graded further; none was reference-positive. D < 0 is not a diagnosis of retrolisthesis, which the readers did not assess (Appendix A). Accuracy is reported for the complete procedure in all 250 segments, with D < 0 counted as a negative result, and for the classification step in the 160 segments with D ≥ 0, contributed by all 50 patients.

### 2.5. Statistical Analysis

To verify the reproducibility of the reference standard, agreement between the two radiologists was quantified before adjudication and reported in accordance with the Guidelines for Reporting Reliability and Agreement Studies (GRRAS) [34]. This analysis concerns the reference standard; the QSA parameters were the index test. The unit of the agreement analysis was the motion segment, and all 250 segments were analysed together. Because a single κ can be misleading when one category dominates, complementary statistics are reported: raw observed agreement with Wilson confidence intervals (CIs); Cohen’s κ [35] with bootstrap confidence intervals (5000 resamples) and the Landis and Koch interpretation [36]; the prevalence-adjusted bias-adjusted κ (PABAK) together with the prevalence and bias indices [37]; Gwet’s first-order agreement coefficient AC1, which is robust to the high-prevalence paradox [34]; positive and negative specific agreement; and the exact McNemar test for directional disagreement between readers.

The motion segment was the unit of analysis; within-patient clustering (up to five segments per patient) was addressed by patient-level cluster bootstrap (2000 resamples, percentile 95% CIs [38]) for all area under the curve (AUC) estimates and AUC differences. QSA uses no information on anatomical level, and the same parameters and thresholds apply at every lumbar level. Incremental value beyond level was assessed by comparing a level-only logistic model with level-plus-QSA models under the same leave-one-patient-out procedure, with paired patient-cluster bootstrap CIs for ΔAUC. Continuous parameters are summarized as median (interquartile range, IQR) and compared with the Mann–Whitney U test, interpreted descriptively. For the classical single-threshold analysis of K, the full receiver operating characteristic (ROC) curve was computed, and operating characteristics were evaluated for 61 candidate thresholds spanning [0.70, 1.00] in steps of 0.005; sensitivity, specificity, positive and negative predictive values (PPV, NPV), likelihood ratios (LR+, LR−), accuracy and the Youden index (J) were tabulated for every candidate, and the optimum was reported both on the full grid and on the grid restricted to one-decimal thresholds. Predictive performance of K was compared with |D|, |F| and |M − 95°| by paired ΔAUC with cluster-bootstrap CIs. Multivariable logistic models (L2-penalized, C = 1) were fitted on standardized predictors. Leave-one-patient-out cross-validation (LOPO-CV) was the primary validation, with standardization and model fitting within each training fold and the AUC computed on pooled out-of-fold predictions; segment-level leave-one-out cross-validation (LOO-CV) is shown for comparison. The candidate predictor sets were specified after inspection of the full sample; a nested analysis repeated this choice within each training fold. Balanced thresholds were selected by the Youden index [39]. Thresholds selected on the full sample are reported as apparent. The development of the two-step rule (Section 3.8) was repeated within each LOPO training set. In each fold, all data-dependent steps used the training patients only: (i) the predictor set was the candidate model with the highest inner leave-one-patient-out AUC within the training set, with ties resolved to the earlier model in the pre-specified order; (ii) the tier boundaries were the lowest K among training reference-negative segments and the highest K among training reference-positive segments; held-out segments with K below the lower boundary were assigned to the rule-in tier, those above the upper boundary to the rule-out tier and all others, including K equal to a boundary, to the intermediate tier, without rounding; (iii) the N cut-off was the Youden optimum among the observed N values of the training set, with ties resolved to the lowest cut-off. Fold-specific results are given in Table S1. Threshold-specific sensitivity, specificity and predictive values carry patient-cluster bootstrap percentile 95% CIs; where no errors were observed, exact Clopper–Pearson limits are given on segments and on patients. Reporting follows the Standards for Reporting of Diagnostic Accuracy Studies (STARD) 2015 [18]. No a priori sample-size calculation was performed. Analyses used Python 3.12.3 (Python Software Foundation, Wilmington, DE, USA) (NumPy 2.4.4, pandas 3.0.2, SciPy 1.17.1, scikit-learn 1.8.0, statsmodels 0.14.6 [40]); α = 0.05, two-sided. Generative AI (Claude, Anthropic; Opus 5 and Opus 5.5; Anthropic, San Francisco, CA, USA) was used to develop and check the analysis code, to run the analyses added during revision, to prepare figures and for language editing; all code, results and text were verified by the authors.

### 2.6. Simulation of Annotation Error

Because the endplate lines were drawn by a single observer, and the radiographs could not be re-read within the approved protocol, the sensitivity of the QSA parameters and classifications to annotation error was assessed by Monte Carlo simulation, an approach used to quantify the effect of landmark placement error on radiographic spinal measurements [41,42]. The quadrilateral of each segment was reconstructed from its exported parameters and scaled to a caudal endplate length of 35 mm; the reconstruction reproduced F, D, K, M and N within their rounding precision in all 160 segments. Each of the four endpoints was then displaced by independent Gaussian errors with a standard deviation of 0.5, 1.0 or 1.5 mm per coordinate; the lowest value corresponds to the precision of corner-based sagittal measurements reported by Frobin et al. [22]. For each scenario, 1000 pairs of simulated readings were generated, and all parameters were recomputed with the pipeline formulas. Agreement between paired readings was summarized by the absolute-agreement intraclass correlation coefficient, ICC(A,1), and the standard error of measurement (SEM), and classification stability by the proportion of segments changing K tier, N class (N ≤ 93°), sign of D or two-step classification.

## 3. Results

### 3.1. Segments and Prevalence

Fifty patients contributed 250 segments; 90 had negative shear (D < 0) and were not graded further, leaving 160 for analysis (Figure 3, Table 2). Anterolisthesis was present in 54 segments (33.8%), located at L4–L5 in 45 cases (83.3%), L3–L4 in 5 (9.3%) and L5–S1 in 4 (7.4%). Every patient contributed at least one positive and at least one negative segment, so each patient served as their own control. As a computational check, the closure identity held within 1.0° in all segments (mean absolute deviation 0.3°).

Global sagittal alignment in the cohort was that of a degenerative, largely compensated lumbar spine. Lumbar lordosis averaged 52.5 ± 9.7° and pelvic incidence 50.6 ± 9.9°, so that the PI–LL mismatch was minimal (median −2.5°, IQR −10.8 to 3.8) and fell within ±10° in 30 patients (60%), while pelvic tilt averaged 18.0 ± 7.0° and exceeded 20° in 22 patients (44%). Lordosis was nevertheless apportioned unevenly between its two arcs—proximal 22.0 ± 7.5° and distal 30.6 ± 7.4°—giving a lordosis distribution index (LDI, lower-arc L4–S1 lordosis as a percentage of total L1–S1 lordosis [17]) of 58.5 ± 11.6% (median 57.7%, range 40.5–84.8). By the established categories, 37 patients (74%) were within the aligned range of 50–80%, 10 (20%) showed moderate hypolordotic maldistribution (40–49%), and 3 (6%) showed hyperlordotic maldistribution (>80%), with none below 40%. The cohort thus combined preserved global balance with a loss of lordosis from the lower arc in one fifth of patients, the alignment pattern expected in degenerative rather than deformity-driven disease.

### 3.2. Reference Standard: Inter-Reader Agreement

Before adjudication, the two readers agreed on 243 of the 250 segments (97.2%, 95% CI 94.3–98.6), with 49 concordant positive and 194 concordant negative verdicts (Table 3). Cohen’s κ was 0.916 (95% CI 0.848–0.974), corresponding to almost perfect agreement [36]. Because negative verdicts predominated (prevalence index 0.580), the prevalence-robust coefficients are reported alongside κ: PABAK 0.944 and Gwet’s AC1 0.958 (0.923–0.988). Specific agreement was 93.3% (86.9–96.7) for positive and 98.2% (96.4–99.1) for negative verdicts.

Reader 1 classified 55 segments as positive, and reader 2 classified 50, with no evidence of systematic directional disagreement (bias index 0.020; exact McNemar *p* = 0.125). The seven discordant segments (2.8%) were adjudicated by the third radiologist, who sided with reader 1 in six and with reader 2 in one; the final reference identified 54 positive segments.

### 3.3. Parameter Distributions

**Figure 4:**
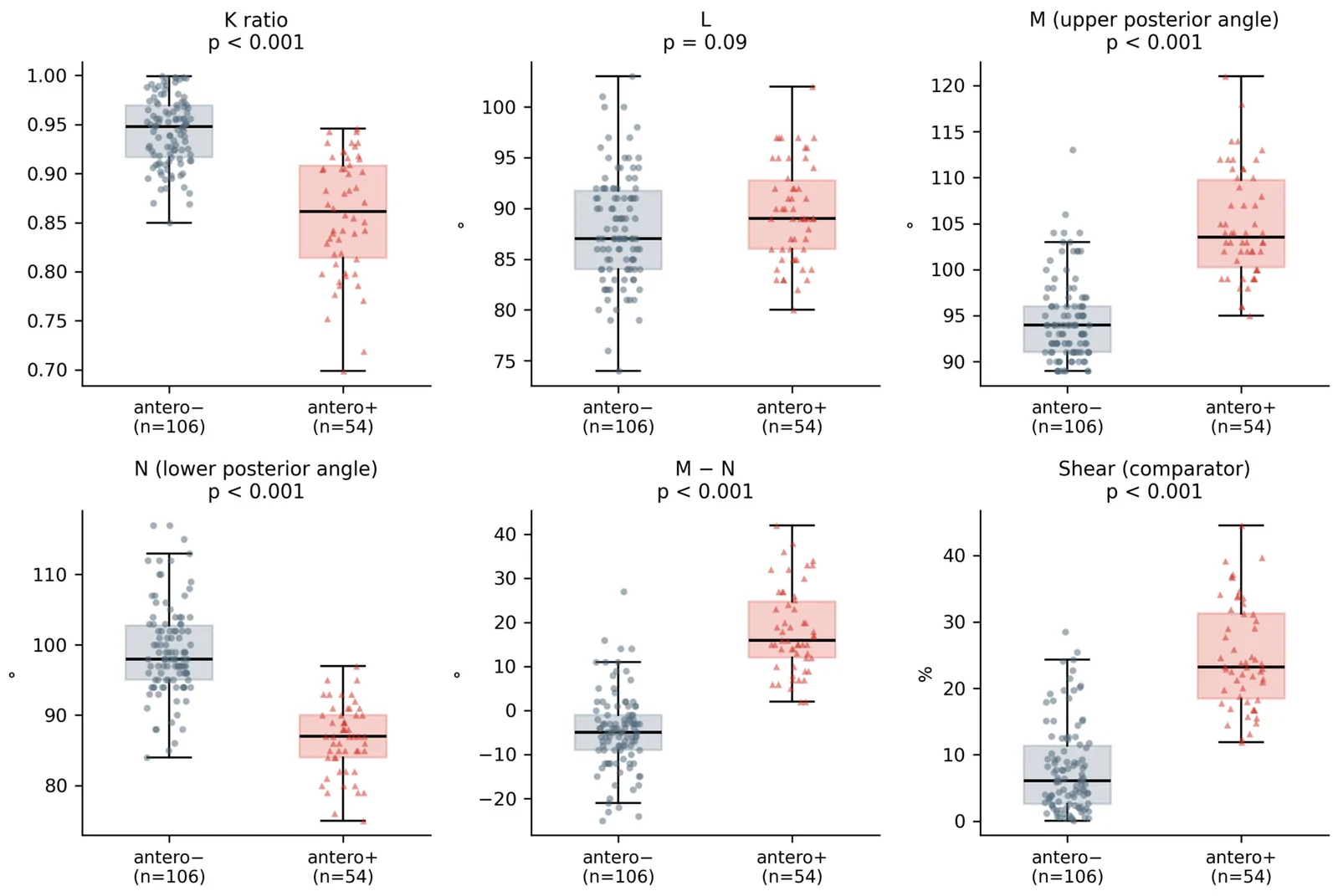
Distributions of the QSA parameters and of segmental shear in reference-negative (grey circles) and reference-positive (red triangles) segments; boxes show median and interquartile range.

All translation-encoding parameters separated the two groups strongly (Table 4, Figure 4). K was lower in anterolisthesis (median 0.861 vs. 0.948), N smaller (87° vs. 98°), M larger (103.5° vs. 94°) and M − N reversed in sign (+16° vs. −5°); |D|, |F| and |M − 95°| were correspondingly increased (all *p* < 0.001). Neither L (*p* = 0.085) nor the rotational and shape descriptors (rotation *p* = 0.091, total rotation *p* = 0.151, wedging *p* = 0.223, tilt *p* = 0.179) differed between groups.

### 3.4. Single-Threshold Discrimination of K

**Figure 5:**
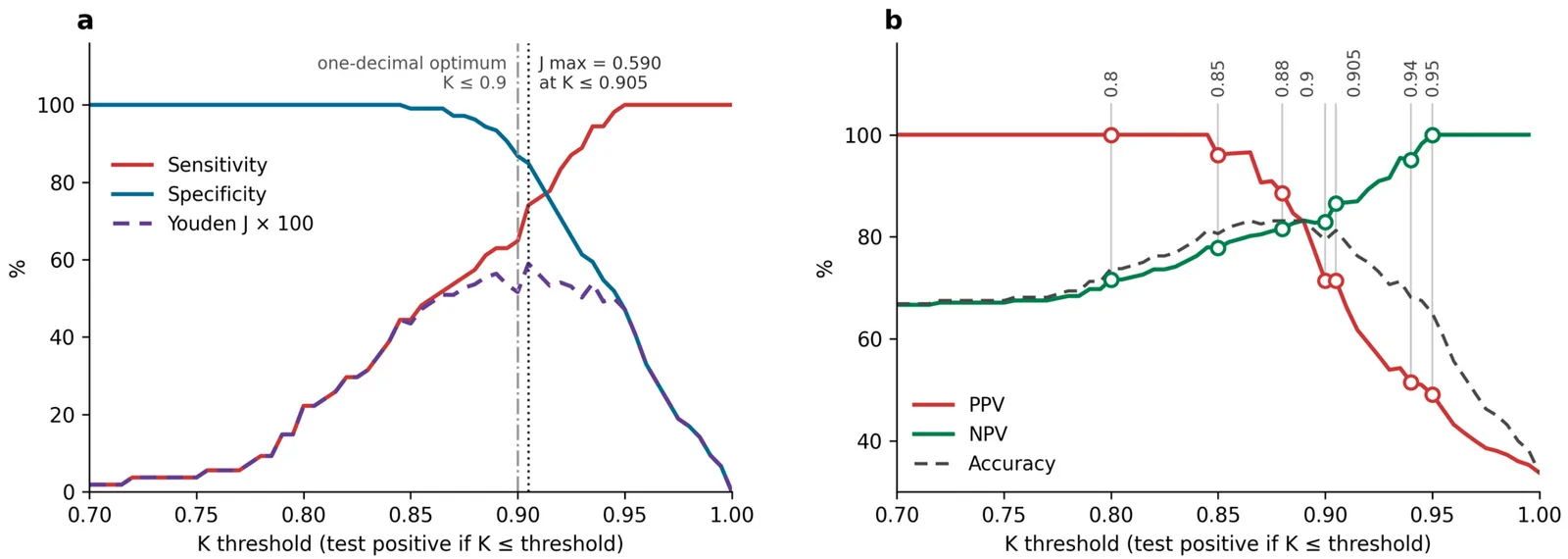
Operating characteristics across the 61 candidate K thresholds. (**a**) Sensitivity, specificity and the Youden index, with the overall optimum (K ≤ 0.905) and the one-decimal optimum (K ≤ 0.9) marked. (**b**) Predictive values and accuracy; circles mark the seven thresholds of Table 5.

K discriminated anterolisthesis with an AUC of 0.892 (95% CI 0.841–0.937). Across the 61 candidate thresholds in [0.70, 1.00], sensitivity rose from 1.9% at K ≤ 0.70 to 100% at K ≤ 0.95, while specificity fell from 100% to 47.2%; the Youden index peaked at K ≤ 0.905 (J = 0.590, sensitivity 74.1%, specificity 84.9%) and displayed a broad plateau between 0.88 and 0.91, so nearby thresholds perform almost identically (Figure 5). When thresholds are restricted to one decimal—the resolution of the three-tier rule (Section 3.8)—the Youden optimum is K ≤ 0.9 (J = 0.516, sensitivity 64.8%, specificity 86.8% and accuracy 79.4%).

Because a single operating point cannot serve every purpose, seven representative thresholds are given in Table 5. The trade-off is monotone and steep at both ends: K ≤ 0.85 supports a slip (specificity 99.1%, PPV 96.0%, LR+ 47.1) at the cost of missing more than half of them, whereas at the cut-off K ≤ 0.95, every reference-positive segment was test-positive (sensitivity 100%); a negative result (K > 0.95) occurred in 50 segments, none reference-positive (NPV 100%, LR− 0.00), at the cost of a specificity of 47.2%.

### 3.5. K Versus Other Geometric Predictors

**Figure 6:**
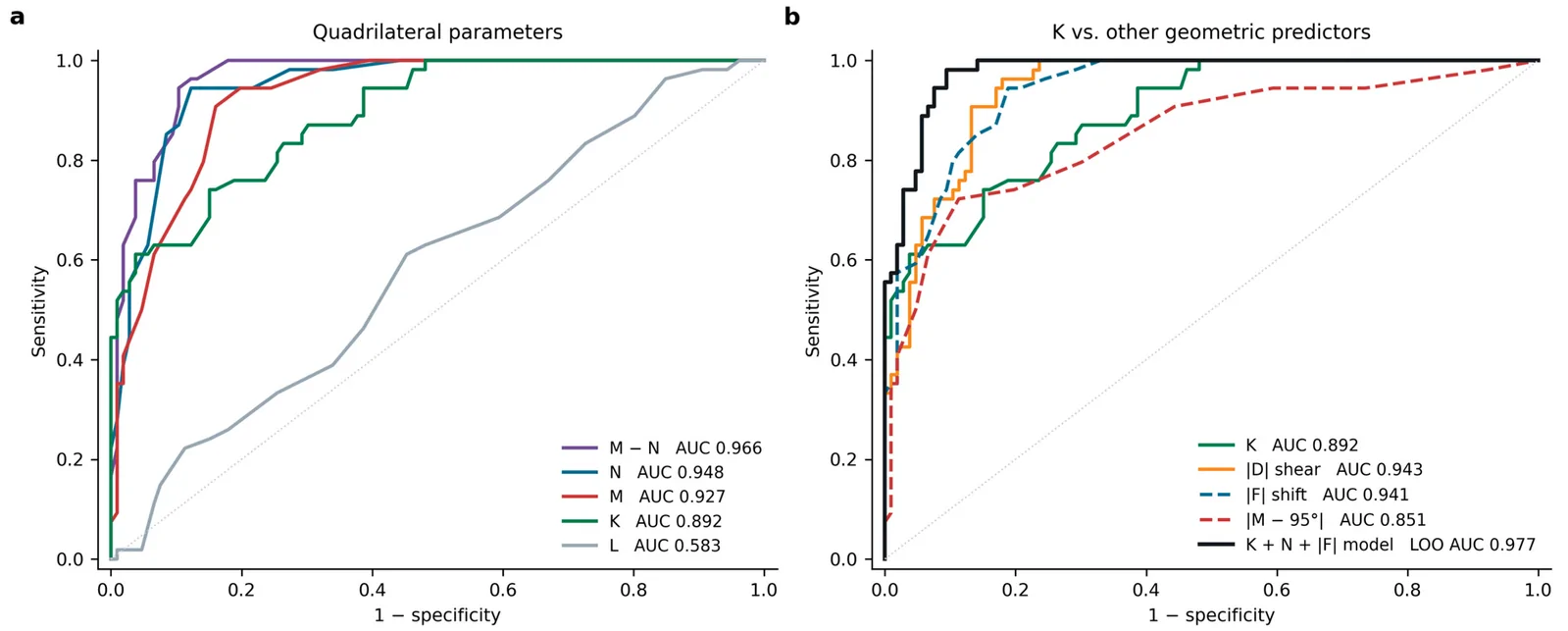
Receiver operating characteristic (ROC) curves. (**a**) QSA parameters. (**b**) K compared with the absolute translation measures |D| and |F|, with |M − 95°|, and with the cross-validated three-parameter model.

K was a good but not the strongest single predictor (Table 6, Figure 6). The direct translation measures |D| (AUC 0.943) and |F| (0.941) and the posterior angles N (0.948) and M − N (0.966) all exceeded it, with cluster-bootstrap ΔAUC excluding zero; the deviation of the upper posterior angle from neutral, |M − 95°| (0.851), was statistically indistinguishable from K (ΔAUC −0.041, 95% CI −0.106 to 0.021). L and the rotational and shape descriptors carried essentially no information (AUC 0.56–0.58; ΔAUC vs. K −0.33 to −0.31), consistent with a translational rather than angular deformity. K correlated only moderately with the translation measures (Spearman ρ = −0.51 with |F|, −0.53 with |D|, −0.56 with |M − 95°|) and weakly with N (−0.35), consistent with its dependence on segmental angle and endplate lengths as well as on translation (Appendix A).

### 3.6. Multivariable Models with Patient-Level Validation

Combining K with a posterior angle produced a marked, cross-validated gain (Table 7). K alone reached a LOPO-CV AUC of 0.888 (95% CI 0.837–0.935); adding N raised it to 0.972 (0.951–0.989), and adding shift as well raised it to 0.978 (0.962–0.991), with segment-level LOO-CV estimates virtually identical. Repeating the choice among the candidate models within each training fold selected K + N + |F| in all 50 folds (LOPO-CV AUC 0.978). At a probability cut-off of 0.5, the K + N model classified segments with 85.2% sensitivity, 95.3% specificity and 91.9% accuracy. Since the increment from the two- to the three-parameter model was small (ΔAUC 0.006), K + N is the parsimonious combination.

### 3.7. Incremental Value Beyond Anatomical Level

Because 45 of the 54 reference-positive segments were at L4–L5, level-only and level-plus-QSA logistic models were compared under the same leave-one-patient-out procedure (Table 8). Level alone reached an AUC of 0.869; at a probability cut-off of 0.5, it reproduced the rule “L4–L5 positive” (sensitivity 83.3%, specificity 95.3%). Adding K, N or M − N to level improved discrimination (ΔAUC 0.095–0.113; all 95% CIs excluded zero), and level, K and N together reached 0.987 (ΔAUC 0.117). K and N without level (0.972) also exceeded level alone. QSA discrimination was therefore not explained by the concentration of anterolisthesis at L4–L5.

### 3.8. Three-Tier Reading Rule

Rounded half-up to one decimal (K_r_), K defined three tiers (Table 9). The rule-in tier (K_r_ < 0.9; K < 0.850) contained 24 segments, all reference-positive (PPV 100%, exact 95% CI 85.8–100), and the rule-out tier (K_r_ = 1.0; K ≥ 0.950) contained 51 segments, none reference-positive (NPV 100%, 93.0–100). Together, 75 of 160 segments (46.9%) were assigned to an extreme tier without observed misclassification (apparent estimate; exact upper 95% confidence limit of the error rate 4.8%). The intermediate tier (K_r_ = 0.9) held 85 segments (53.1%) with a 35.3% slip rate, within which N discriminated well (AUC 0.931). Applying N ≤ 93°, the Youden-optimal cut-off for N in the full sample, in that tier gave a sensitivity of 94.4%, specificity of 95.3% and accuracy of 95.0% (95% CI 91.7–98.1), improving on N alone by 5.0 percentage points of accuracy (cluster bootstrap 1.9–8.6; McNemar *p* = 0.008, 8 errors corrected, none introduced). All eight residual errors fell inside the intermediate tier. With D < 0 counted as a negative index-test result, the complete procedure in all 250 segments yielded 51 true-positive, 3 false-negative, 5 false-positive and 191 true-negative results (prevalence 21.6%): sensitivity 94.4% (95% CI 87.3–100), specificity 97.4% (95.4–99.5), PPV 91.1% (84.2–98.0), NPV 98.5% (96.5–100) and accuracy 96.8% (94.8–98.8). Repeating the development of the rule within LOPO-CV, with the tier boundaries and the N cut-off re-derived in each training set, selected N ≤ 93° in all 50 folds and gave identical estimates. A three-tier rule based on N, with boundaries at the class extremes (N < 84° and N > 97°), assigned 71 of 160 segments (44.4%) to extreme tiers without observed misclassification.

### 3.9. Sensitivity to Annotation Error

Simulated endpoint error reduced agreement in proportion to its size (Table 10). With a standard deviation of 0.5 mm, ICC(A,1) was 0.92 for K, 0.96 for N, 0.95 for M − N and 0.93 for F; with 1.0 mm, it was 0.74, 0.85, 0.83 and 0.78. The SEM of K, 0.017 at 0.5 mm and 0.033 at 1.0 mm, was a substantial fraction of the 0.1-wide intermediate band, so the K tier changed in 19% and 38% of segments. The two-step classification changed less often, in 5% and 13% of segments. Under simulated re-annotation, the accuracy of the two-step rule was 92.5% at 0.5 mm and 89.4% at 1.0 mm, compared with 95.0% for the original annotation.

## 4. Discussion

### 4.1. Principal Findings

QSA turns a translation problem into an angular one. Anterior displacement skews the segmental quadrilateral, so the posterior angles and the diagonal ratio encode the slip without distance measurement. In patients with established degenerative spondylolisthesis, these calibration-free descriptors discriminated as well as percentage translation computed from the same lines. L and all rotational and wedging descriptors were uninformative. This contrast is partly built into the design, because the reference standard defines anterolisthesis by translation. It is consistent, however, with flexion–extension data in which translation, rather than angulation, was related to symptoms [43]. The standing film records the position of the segment under axial load, and QSA quantifies it. Unlike flexion–extension imaging, it requires no effort from the patient; in one series, detected instability rose from 6% to 38% after analgesia [44].

### 4.2. Decision Thresholds

The threshold sweep argues against a single cut-off for K. The Youden optimum (0.905) is a compromise; thresholds of potential clinical interest lie at the extremes, 0.85 to support and 0.95 to argue against a slip. Rounded to one decimal, the same parameter yields a three-tier rule that assigned 75 of 160 segments (47%) to an extreme tier without observed misclassification (Table 9). All 24 segments in the positive tier were reference-positive (PPV 100%; exact 95% CI 85.8–100), and all 51 in the negative tier were reference-negative (NPV 100%; exact 95% CI 93.0–100). Zero observed errors remain compatible with a true error rate of up to 4.8% [45]. These estimates are apparent, because the tier boundaries coincide with the class extremes of this sample; nested validation gave identical results (Section 3.8). The 85 intermediate segments were passed to a second step (N ≤ 93°). Reporting them in a 3 × 2 classification, rather than discarding them, preserves this information [46]. The cross-validated K + N model (AUC 0.972) shows that the two descriptors are complementary: K measures diagonal asymmetry regardless of direction, whereas N is signed. A three-tier rule based on N performed similarly (71 vs. 75 segments in extreme tiers; Section 3.8); rounding by itself does not establish clinical utility.

### 4.3. Comparison with Existing Methods

QSA avoids the scaling dependence of millimetre thresholds. It shares scale independence with slip percentage and with endplate-normalized motion metrics [11]. It differs from both by deriving the slip-percentage analogue F, the posterior angles M and N and the diagonal ratio K from a single construction with exact relations between them (Appendix A). The posterior angles do not require the posteroinferior corner, and N and M − N discriminated similarly to |F| (Table 6). Whether this offers a practical advantage over conventional slip percentage, in reliability, automation or dynamic imaging, remains to be tested. Deep-learning detectors reach a pooled sensitivity of 94.7% and specificity of 97.1%, with substantial heterogeneity [47]. A segmentation pipeline published in this journal reported accuracies of 96.1% and 94.4% on internal and external data [48]. QSA reaches comparable discrimination without a trained network, although cross-study comparisons are indirect. Because it uses the endplates and corners that such networks already extract, QSA can serve as an interpretable decision layer on top of automated landmarking.

### 4.4. Clinical Implications

QSA is intended as an add-on to the routine standing radiograph. The positive tier supports anterolisthesis, the negative tier argues against it, and intermediate segments warrant a second parameter or dynamic imaging. Slip thresholds drive several decisions. Appropriate use criteria separate static from dynamic spondylolisthesis when choosing between decompression and fusion [6]. Trials of basivertebral nerve ablation excluded slips greater than 2 mm [49]. Given lumbar magnification of 1.09–1.63 [10], an uncorrected true 2 mm slip may measure 2.2–3.3 mm. QSA could also provide an objective static input to instability classifications [12,50]. It requires six lines per patient, no calibration and no landmark other than the endplate.

The magnitude of slip does not by itself determine the severity of neural compression, which also depends on canal and foraminal dimensions, facet and ligamentum flavum hypertrophy and disc bulging, all of which are assessed on MRI or CT; QSA does not replace this assessment. Nor does QSA characterize vertebral body height, disc height or anterior–posterior disc asymmetry separately. These features nevertheless enter its measurements: the posterior side of the quadrilateral comprises posterior vertebral body height and posterior disc height, disc wedging contributes to α, and K depends on both (Appendix A, Equation (A7)). Marking the inferior endplates as well would separate the vertebral body from the disc and yield calibration-free disc height and disc asymmetry ratios. In practice, QSA can complement these assessments by quantifying the static translation on the same radiograph on which disc height and alignment are judged.

On flexion–extension or standing–supine pairs, ΔN and ΔK would quantify the dynamic component of instability. This extension requires standardized acquisition, because instability classification varies with imaging protocol and pain control [13,44]. It also requires validation against outcomes. In NORDSTEN-DS, neither established instability criteria nor surgeons’ recommendations identified patients who benefited from fusion [14,15].

### 4.5. Limitations

This study has several strengths: a prospectively enrolled cohort, a predefined criterion-based reference standard with blinded double reading and adjudication and an index test that is calibration-free by construction and transparent at every step.

All participants had degenerative spondylolisthesis, and every patient contributed both reference-positive and reference-negative segments. The estimates therefore describe discrimination between segments within affected patients, not diagnostic performance in an unselected population assessed for suspected disease, in which prevalence, case mix and the proportion of borderline slips differ. Reference-positive segments were concentrated at L4–L5 (45 of 54), as is typical of degenerative spondylolisthesis, and level-specific accuracy could not be estimated reliably.

This is a single-centre analysis with a case mix typical of degenerative spondylolisthesis, in which 90% of L4–L5 segments were reference-positive; at lower prevalence, with sensitivity and specificity unchanged, the positive predictive value would be lower and the negative predictive value higher. Segments are nested within patients, and intervals that ignore this clustering overstate precision [51]; likewise, segment-level leave-one-out cross-validation allows segments from the same patient to inform both training and testing, so leave-one-patient-out validation, which better approximates prospective use, was the primary scheme [52].

The reference standard was expert visual judgement: criterion-based, double-read (κ 0.916) and adjudicated; it was not verified against dynamic imaging or computed tomography, so part of the residual disagreement—four of the five false positives showed geometric translation above 15%—may reflect the limits of the reference rather than those of the index test. Because the reference and the index test read the same radiograph, and the reference includes direct translation criteria, some shared information is unavoidable and may favour translational descriptors.

Above all, a single film captures only the static component of instability: QSA quantifies the loaded position, not motion, and its dynamic extension remains untested. Scale independence also does not imply projection independence: axial rotation, beam obliquity and double endplate contours, as well as irregular endplates, transitional anatomy and pelvic overlap at L5–S1, can affect line placement. In degenerative scoliosis, axial rotation and lateral translation accompany anterolisthesis; the lateral projection then no longer shows single endplate contours, and QSA, like other measurements on a lateral radiograph, may misrepresent the sagittal slip. Patients with deformity were excluded, so QSA should not be applied to them without validation, preferably against biplanar or three-dimensional imaging.

The principal limitation is that the reproducibility of the endplate annotation, drawn by a single observer, has not been tested with repeated readings. The simulation (Section 3.9) indicates that K tier assignment is sensitive to millimetric endpoint error, whereas N and the two-step classification are more robust; it cannot, however, capture systematic differences between observers, such as different interpretations of endplate rims, although landmark labelling on lumbar radiographs following a written procedure has shown high inter-rater reliability [53]. All thresholds are therefore provisional, and a blinded reproducibility study following GRRAS recommendations [34], with absolute-agreement intraclass correlation coefficients (ICCs) [54], measurement error and tier stability relative to the 0.1-wide intermediate band, is the first step of the planned validation. Thresholds derived here, including the three-tier boundaries, are data-driven, an approach known to inflate accuracy estimates, particularly in small samples [55]; they are reported as apparent and need external validation. Segments with negative shear (n = 90) were not graded. Because D depends on endplate length and segmental angle, D < 0 does not identify retrolisthesis, and performance in retrolisthesis remains to be established. K close to 1 does not exclude a small slip in a lordotic segment (Appendix A); with the smallest reference-positive slip at F = 9%, the rule-out tier has not been tested for small or borderline slips. Finally, deep-learning systems now detect spondylolisthesis and quantify slip on lateral radiographs automatically [48,56]; using their landmarks as input to QSA would remove the single-observer limitation but remains to be tested.

## 5. Conclusions

Quadrilateral segmental analysis detected degenerative lumbar anterolisthesis, the static radiographic component of segmental instability, on a single standing lateral radiograph without distance calibration.

The diagonal ratio K translates the radiologist’s assessment of anterolisthesis into a single number. It discriminated well (AUC 0.892), with purpose-dependent thresholds: 0.85 to support a slip, 0.9 for balanced reading and 0.95 to argue against it. Rounded to one decimal, K assigned nearly half of all segments to extreme tiers without observed misclassification. The rule-out tier does not establish performance for small or borderline slips, because the smallest reference-positive slip was 9% and K close to 1 can coexist with a small slip in a lordotic segment. The intermediate tier was resolved by the lower posterior angle N, and K combined with N reached a cross-validated AUC of 0.972. QSA requires no calibration and is suited to full automation. These exploratory findings, based on thresholds derived in a single sample annotated by a single observer, require replication in independent cohorts and by other observers before clinical application; extension to dynamic image pairs is needed for a complete assessment of instability.

## Figures and Tables

**Table 1:** Demographic and radiographic characteristics of the study cohort (50 patients and 250 motion segments).

Characteristic	Value
**Patients (n = 50)**	
Age, years	68.4 ± 8.0 (46 to 81)
Female sex, n (%)	34 (68.0)
**Sagittal alignment, standing lateral radiograph**	
Lumbar lordosis (LL, L1–S1), °	52.5 ± 9.7 (24 to 69)
Proximal lumbar lordosis (PLL, L1–L4), °	22.0 ± 7.5 (5 to 38)
Distal lumbar lordosis (DLL, L4–S1), °	30.6 ± 7.4 (10 to 44)
Lordosis distribution index (LDI), % ^a^	58.5 ± 11.6 (40.5 to 84.8)
<50% (hypolordotic maldistribution), n (%)	10 (20.0)
50–80% (aligned), n (%)	37 (74.0)
>80% (hyperlordotic maldistribution), n (%)	3 (6.0)
Pelvic incidence (PI), °	50.6 ± 9.9 (32 to 69)
Pelvic tilt (PT), ° ^b^	18.0 ± 7.0 (−1 to 30)
Sacral slope (SS), °	32.6 ± 7.1 (21 to 49)
PI − LL mismatch, °	−1.9 ± 10.4 (−27 to 23)
PI − LL > 10°, n (%)	7 (14.0)
**Motion segments, reference standard classification**	
Segments assessed (L1–L2 to L5–S1), n ^c^	250
D < 0, not graded further, n	90
D ≥ 0, included in the accuracy analysis, n	160
Reference-positive (anterolisthesis), n/N (%)	54/160 (33.8)
L1–L2	0/7 (0.0)
L2–L3	0/16 (0.0)
L3–L4	5/38 (13.2)
L4–L5	45/50 (90.0)
L5–S1	4/49 (8.2)
Patients by number of reference-positive levels, n (%)	
One level	46 (92.0)
Two adjacent levels ^d^	4 (8.0)
Shift (F) in reference-positive segments, % ^e^	23 (17–30; range 9–43) –

Values are mean ± SD (range) or n (%) unless otherwise indicated. Bold rows are section headings. ^a^ LDI = DLL/LL × 100; categories according to Yilgor et al. [17]. ^b^ Mildly non-normal distribution (Shapiro–Wilk *p* = 0.04); median 18.5° (IQR 15.0–23.0°). ^c^ Segment-level classification by the adjudicated two-reader reference standard; segments with D < 0 were not graded further (Section 2.4), and none of them was reference-positive. ^d^ L3–L4 with L4–L5 (n = 2) and L4–L5 with L5–S1 (n = 2). ^e^ Median (IQR; range); F as defined in Section 2.3. Abbreviations: DLL, distal lumbar lordosis; IQR, interquartile range; LDI, lordosis distribution index; LL, lumbar lordosis; PI, pelvic incidence; PLL, proximal lumbar lordosis; PT, pelvic tilt; SD, standard deviation; SS, sacral slope.

**Table 2:** Segment flow and prevalence of anterolisthesis by level.

Level	Segments Assessed	D < 0	D ≥ 0 (Analysed)	Anterolisthesis, n (%)
L1–L2	50	43	7	0 (0.0)
L2–L3	50	34	16	0 (0.0)
L3–L4	50	12	38	5 (13.2)
L4–L5	50	0	50	45 (90.0)
L5–S1	50	1	49	4 (8.2)
Total	250	90	160	54 (33.8)

D < 0: negative segmental shear; these segments were not graded further (Section 2.4). All reference-positive segments had D ≥ 0.

**Table 3:** Agreement between the two independent radiologists before adjudication (250 motion segments).

Statistic	Value (95% CI)	Interpretation
Concordant verdicts, positive/negative	49/194	243 of 250 segments
Discordant verdicts, reader 1+/reader 2+	6/1	7 segments (2.8%) adjudicated
Observed agreement	97.2% (94.3–98.6)	—
Cohen’s κ	0.916 (0.848–0.974)	Almost perfect
PABAK	0.944	Prevalence-adjusted
Gwet’s AC1	0.958 (0.923–0.988)	Robust to prevalence
Positive specific agreement	93.3% (86.9–96.7)	—
Negative specific agreement	98.2% (96.4–99.1)	—
Prevalence index/bias index	0.580/0.020	Low bias
McNemar test (exact)	*p* = 0.125	No systematic bias

Wilson confidence intervals for proportions; bootstrap (5000 resamples) for κ and AC1. PABAK—prevalence-adjusted bias-adjusted kappa.

**Table 4:** QSA parameters and comparators by reference standard, median (IQR).

Parameter	Anterolisthesis (n = 54)	No Anterolisthesis (n = 106)	*p* *
K—diagonal ratio	0.861 (0.814–0.908)	0.948 (0.917–0.970)	<0.001
M—upper posterior angle (°)	103.5 (100.3–109.8)	94 (91–96)	<0.001
N—lower posterior angle (°)	87 (84–90)	98 (95–102.8)	<0.001
M − N (°)	+16 (12–24.8)	−5 (−9 to −1)	<0.001
|M − 95°| (°)	8.5 (5.3–14.8)	3 (1–5)	<0.001
L—inter-diagonal angle (°)	89 (86–92.8)	87 (84–91.8)	0.085
|D|—shear (%)	23.3 (18.4–31.2)	6.1 (2.6–11.4)	<0.001
|F|—shift (%)	23 (17.3–30)	5.5 (2–10)	<0.001
Segmental rotation α (°)	11 (9–15)	12 (9–19)	0.091
Wedging (%)	21 (15–27)	23 (15.3–35.5)	0.223
Tilt (°)	−1.5 (−7.8 to 3)	0 (−10 to 12)	0.179

* Mann–Whitney U test, unadjusted for within-patient clustering (descriptive).

**Table 5:** Operating characteristics of seven candidate K thresholds (test positive if K ≤ threshold; apparent estimates; patient-cluster bootstrap 95% CIs).

Threshold	Intended Use	Se, % (95% CI)	Sp, % (95% CI)	PPV, % (95% CI)	NPV, % (95% CI)	J	LR+/LR−	Acc, %
K ≤ 0.80	Maximum specificity	22.2 (10.9–35.2)	100 (96.6–100) *	100 (73.5–100) *	71.6 (66.5–76.3)	0.222	∞/0.78	73.8
K ≤ 0.85	Rule-in	44.4 (32.1–56.9)	99.1 (96.9–100)	96.0 (85.7–100)	77.8 (72.5–82.9)	0.435	47.1/0.56	80.6
K ≤ 0.88	Rule-in, higher yield	57.4 (45.5–69.8)	96.2 (92.3–99.1)	88.6 (78.1–97.3)	81.6 (76.4–86.9)	0.536	15.2/0.44	83.1
K ≤ 0.90	One-decimal optimum	64.8 (52.8–76.9)	86.8 (80.4–92.7)	71.4 (61.0–82.9)	82.9 (77.3–88.7)	0.516	4.91/0.41	79.4
K ≤ 0.905	Youden optimum	74.1 (61.5–85.2)	84.9 (78.4–91.3)	71.4 (62.1–81.7)	86.5 (81.2–91.9)	0.590	4.91/0.31	81.2
K ≤ 0.94	Screening (rule-out)	94.4 (87.7–100)	54.7 (44.5–64.9)	51.5 (45.1–59.2)	95.1 (89.1–100)	0.492	2.09/0.10	68.1
K ≤ 0.95	Maximum sensitivity	100 (93.4–100) *	47.2 (37.4–57.4)	49.1 (43.7–55.6)	100 (92.9–100) *	0.472	1.89/0.00	65.0

Se—sensitivity; Sp—specificity; PPV/NPV—predictive values at the observed prevalence (33.8%); J—Youden index; LR—likelihood ratio; Acc—accuracy. Thresholds were selected on the full sample; K was recorded to three decimals. * Exact Clopper–Pearson interval (no errors observed). Counts (TP/FN/FP/TN): K ≤ 0.80, 12/42/0/106; K ≤ 0.85, 24/30/1/105; K ≤ 0.88, 31/23/4/102; K ≤ 0.90, 35/19/14/92; K ≤ 0.905, 40/14/16/90; K ≤ 0.94, 51/3/48/58; K ≤ 0.95, 54/0/56/50.

**Table 6:** Discrimination of single geometric predictors, with paired comparison against K.

Predictor	AUC (95% CI)	ΔAUC vs. K (95% CI)	Interpretation
M − N (°)	0.966 (0.944–0.985)	+0.074 (+0.030 to +0.126)	Superior to K
N (°)	0.948 (0.919–0.975)	+0.056 (+0.016 to +0.107)	Superior to K
|D|—shear (%)	0.943 (0.909–0.968)	+0.050 (+0.003 to +0.105)	Superior to K
|F|—shift (%)	0.941 (0.910–0.966)	+0.049 (+0.001 to +0.105)	Superior to K
M (°)	0.927 (0.892–0.956)	+0.035 (−0.018 to +0.095)	Comparable
K—diagonal ratio	0.892 (0.841–0.937)	reference	—
|M − 95°| (°)	0.851 (0.787–0.905)	−0.041 (−0.106 to +0.021)	Comparable
L (°)	0.583 (0.506–0.661)	−0.309 (−0.401 to −0.210)	Non-discriminative
Segmental rotation α (°)	0.582 (0.504–0.659)	−0.311 (−0.404 to −0.219)	Non-discriminative
Total rotation (%)	0.570 (0.487–0.647)	−0.323 (−0.420 to −0.230)	Non-discriminative
Wedging (%)	0.559 (0.478–0.635)	−0.333 (−0.426 to −0.238)	Non-discriminative
Tilt (°)	0.565 (0.493–0.630)	−0.327 (−0.403 to −0.255)	Non-discriminative

AUCs of the rotational and shape descriptors are reported with the orientation that maximizes discrimination, and ΔAUC was computed from these oriented AUCs. ΔAUC from 2000 patient-cluster bootstrap resamples.

**Table 7:** Multivariable logistic models of anterolisthesis with internal validation.

Model	Apparent AUC	LOO-CV AUC (95% CI)	LOPO-CV AUC (95% CI)	Se/Sp at *p* = 0.5, %	Accuracy, %
K	0.892	0.886 (0.833–0.934)	0.888 (0.837–0.935)	63.0/93.4	83.1
K + |M − 95°|	0.909	0.898 (0.849–0.940)	0.901 (0.856–0.941)	64.8/92.5	83.1
K + |F|	0.965	0.959 (0.933–0.980)	0.961 (0.936–0.981)	74.1/95.3	88.1
K + N	0.977	0.972 (0.951–0.990)	0.972 (0.951–0.989)	85.2/95.3	91.9
K + N + |M − 95°|	0.977	0.971 (0.950–0.989)	0.972 (0.952–0.989)	85.2/94.3	91.2
K + N + |F|	0.981	0.977 (0.960–0.992)	0.978 (0.962–0.991)	83.3/94.3	90.6

L2-penalized logistic regression (C = 1); standardization and fitting within each training fold. LOPO-CV is the primary estimate, and LOO-CV is shown for comparison; sensitivity, specificity and accuracy are from LOPO-CV out-of-fold predictions. LOO-CV—leave-one-out cross-validation (segment level); LOPO-CV—leave-one-patient-out cross-validation; CIs from patient-cluster bootstrap of out-of-fold predictions.

**Table 8:** Level-only and level-plus-QSA logistic models under leave-one-patient-out cross-validation.

Model	AUC (95% CI)	ΔAUC vs. Level Only (95% CI)
Level only	0.869 (0.786–0.946)	—
Level + K	0.964 (0.937–0.987)	+0.095 (0.034 to 0.161)
Level + N	0.977 (0.954–0.995)	+0.108 (0.045 to 0.177)
Level + M − N	0.982 (0.966–0.995)	+0.113 (0.045 to 0.183)
Level + K + N	0.987 (0.973–0.998)	+0.117 (0.049 to 0.192)
K + N (without level)	0.972 (0.951–0.989)	+0.102 (0.023 to 0.184)

Level entered as indicators for L3–L4, L4–L5 and L5–S1 (reference: L1–L3, without reference-positive segments). L2-penalized logistic regression (C = 1); standardization and fitting within each training fold. AUC from pooled out-of-fold predictions; 95% CIs from 2000 patient-cluster bootstrap resamples, paired for ΔAUC.

**Table 9:** Three-tier rule based on K rounded half-up to one decimal (K_r_); apparent estimates in the 160 segments with D ≥ 0.

Tier	Raw K	n (%)	Anterolisthesis	Post-Test Probability (95% CI)	Action
K_r_ < 0.9	< 0.850	24 (15.0)	24/24	100% (85.8–100)	Rule in
K_r_ = 0.9	0.850 ≤ K < 0.950	85 (53.1)	30/85	35.3% (27.1–44.2)	Apply N ≤ 93°
K_r_ = 1.0	≥ 0.950	51 (31.9)	0/51	0% (0–7.0)	Rule out

K_r_, K rounded half-up to one decimal; raw intervals: K_r_ < 0.9, K < 0.850; K_r_ = 0.9, 0.850 ≤ K < 0.950; K_r_ = 1.0, K ≥ 0.950 (K recorded to three decimals). Exact Clopper–Pearson 95% CIs for the extreme tiers (on patients: rule-in, 24 patients, 85.8–100; rule-out, 34 patients, 0–10.3); patient-cluster bootstrap CI for the intermediate tier. Two-step rule (K tiers, then N ≤ 93° in the intermediate tier): sensitivity 94.4%, specificity 95.3%, PPV 91.1%, NPV 97.1%, accuracy 95.0%; identical under nested LOPO-CV.

**Table 10:** Sensitivity of QSA parameters and classifications to simulated annotation error (1000 pairs of simulated readings per scenario).

Endpoint SD, mm	ICC K	ICC N	ICC M − N	ICC F	SEM K	K-Tier Change, %	Two-Step Change, %	Accuracy, %
0.5	0.92	0.96	0.95	0.93	0.017	19.4	5.0	92.5
1.0	0.74	0.85	0.83	0.78	0.033	37.5	12.5	89.4
1.5	0.56	0.72	0.69	0.61	0.045	48.8	19.4	85.0

SD, standard deviation of the displacement of each endpoint per coordinate (radial root-mean-square error 0.71, 1.41 and 2.12 mm) for a caudal endplate length of 35 mm. ICC, absolute-agreement intraclass correlation coefficient ICC(A,1) between paired simulated readings; SEM, standard error of measurement. Values are medians of 1000 simulations. Accuracy of the two-step rule against the reference standard in the 160 segments with D ≥ 0 (original annotation: 95.0%).

## Data Availability

The de-identified segment-level data and the analysis code presented in this study are available on request from the corresponding author. The data are not publicly available due to privacy restrictions and because the PROGRES cohort study is ongoing.

## Supplementary Materials

The following supporting information can be downloaded at: https://www.mdpi.com/article/10.3390/diagnostics16203291/s1. Checklist S1: STROBE checklist for cohort studies; Checklist S2: STARD 2015 checklist; File S1: Reader instructions for the reference standard and endpoint-placement rules; Table S1: Fold-specific tier boundaries, N cut-offs and selected predictor sets in leave-one-patient-out validation.

## Abbreviations

The following abbreviations are used in this manuscript:

AC1	Gwet’s first-order agreement coefficient
AUC	area under the curve
CI	confidence interval
DICOM	Digital Imaging and Communications in Medicine
DLL	distal lumbar lordosis
GRRAS	Guidelines for Reporting Reliability and Agreement Studies
ICC	intraclass correlation coefficient
IQR	interquartile range
LDI	lordosis distribution index
LL	lumbar lordosis
LOO-CV	leave-one-out cross-validation
LOPO-CV	leave-one-patient-out cross-validation
LR	likelihood ratio
NPV	negative predictive value
PABAK	prevalence-adjusted bias-adjusted kappa
PI	pelvic incidence
PLL	proximal lumbar lordosis
PPV	positive predictive value
PT	pelvic tilt
QSA	quadrilateral segmental analysis
ROC	receiver operating characteristic
SD	standard deviation
SS	sacral slope
STARD	Standards for Reporting of Diagnostic Accuracy Studies
STROBE	Strengthening the Reporting of Observational Studies in Epidemiology

## Appendix A. Geometry of the QSA Construction

The caudal endplate line is the *x*-axis, anterior is positive, the lower posterior endpoint P_inf_ is the origin, and the upper posterior endpoint is P_sup_ = (t, H). α is the angle between the endplate lines (lordosis positive); w_sup_ and w_inf_ are the lengths of the cranial and caudal endplate lines; P = |P_sup_P_inf_| is the posterior side of the quadrilateral; d_1_ = |A_inf_P_sup_| and d_2_ = |P_inf_A_sup_| are its diagonals. Positive F and D denote anterior translation of the cranial vertebra. Then:

N = 90° − arctan(t/H) (A1)

M = 90° + α + arctan(t/H) (A2)

M + N = 180° + α (A3)

M − N = α + 2·arctan(t/H) (A4)

F = 100·(t + H·tan α)/w_inf_ (A5)

D = ρF + 50(1 − ρ), with ρ = (w_inf_/w_sup_)·cos α (A6)

d_1_^2^ = w_inf_^2^ + P^2^ − 2w_inf_P·cos N;  d_2_^2^ = w_sup_^2^ + P^2^ − 2w_sup_P·cos M (A7)

K_dir_ = d_1_/d_2_;  K = min(K_dir_, 1/K_dir_) (A8)

F is the distance along the caudal endplate line from P_inf_ to the point where the perpendicular to the cranial endplate line through P_sup_ meets it, as a percentage of caudal endplate length; it equals the conventional slip percentage when the posterior wall of the cranial vertebra is perpendicular to its superior endplate. D is the displacement between the endplate midpoints, projected on the cranial endplate line, as a percentage of its length. By Equation (A6), D contains a term independent of translation, so D < 0 can occur without posterior translation when ρ > 1.

Without translation (F = 0), M = 90° and N = 90° + α exactly. With equal endplate lengths w, the diagonals are equal only when M = N, and without translation K = √[(P^2^ + w^2^)/(P^2^ + w^2^ + 2Pw·sin α)], which is below 1 in every lordotic segment (about 0.91 for P = 0.8w and α = 12°). With increasing anterior translation, K first rises to 1 (at F ≈ 8% in this example) and then falls, whereas K_dir_ decreases monotonically.

If the posterior wall of the cranial vertebra deviates by δ from the normal to its superior endplate (δ > 0 when its caudal end lies anterior to the normal), the slip percentage based on the extended posterior wall is F_wall_ = 100·[t + H·tan(α + δ)]/w_inf_. Then,

F − F_wall_ = 100·H·[tan α − tan(α + δ)]/w_inf_;  if F_wall_ = 0: N = 90° + α + δ, M = 90° − δ (A9)

For typical lumbar proportions (H ≈ 0.8–1.0 w_inf_), F changes by about 1.4–1.8 percentage points per degree of δ.
