# Supplementary material for: Quadrilateral Segmental Analysis (QSA): A Calibration-Free Geometric Method for Detecting Lumbar Spondylolisthesis
Source: Diagnostics (Basel). 2026 Oct 10;16(20):3291. doi: 10.3390/diagnostics16203291 (PMC13703141; doi:10.3390/diagnostics16203291)
Supplement: Supplementary file 1 [file diagnostics-16-03291-s001.zip › QSA_Checklist_STARD.pdf]

Supplementary Material

Quadrilateral Segmental Analysis (QSA): A Calibration-Free Geometric Method for Detecting Lumbar Spondylolisthesis

Szczepański T., Jasiński M., Koźba-Gosztyła M., Więcław J., Szymczyk P., Bladowska J., Czapiga B.

STARD 2015 checklist

Applies to the diagnostic accuracy analysis reported in this manuscript. Section numbers refer to the submitted manuscript.

No.	Item	Reported in
**TITLE OR ABSTRACT**		
1	Identification as a study of diagnostic accuracy using at least one measure of accuracy (such as sensitivity, specificity, predictive values, or AUC)	Title; Abstract (Results)
**ABSTRACT**		
2	Structured summary of study design, methods, results, and conclusions (for specific guidance, see STARD for Abstracts)	Abstract
**INTRODUCTION**		
3	Scientific and clinical background, including the intended use and clinical role of the index test	Section 1, paragraphs 1–4
4	Study objectives and hypotheses	Section 1, final paragraph
**METHODS — Study design**		
5	Whether data collection was planned before the index test and reference standard were performed (prospective study) or after (retrospective study)	Section 2.1, first sentence (retrospective analysis of prospectively collected data)
**METHODS — Participants**		
6	Eligibility criteria	Section 2.1, paragraph 2
7	On what basis potentially eligible participants were identified (such as symptoms, results from previous tests, inclusion in registry)	Section 2.1, paragraph 2
8	Where and when potentially eligible participants were identified (setting, location and dates)	Section 2.1, paragraphs 1–2
9	Whether participants formed a consecutive, random or convenience series	Section 2.1, paragraph 2 (consecutive)
**METHODS — Test methods**		
10a	Index test, in sufficient detail to allow replication	Section 2.3; Figure 2
10b	Reference standard, in sufficient detail to allow replication	Section 2.2, paragraph 2
11	Rationale for choosing the reference standard (if alternatives exist)	Section 2.2, paragraph 2
12a	Definition of and rationale for test positivity cut-offs or result categories of the index test, distinguishing pre-specified from exploratory	Section 2.5; Section 4.5 (all cut-offs exploratory / data-driven)
12b	Definition of and rationale for test positivity cut-offs or result categories of the reference standard, distinguishing pre-specified from exploratory	Section 2.2, paragraph 2 (pre-specified criteria)
13a	Whether clinical information and reference standard results were available to the performers/readers of the index test	Section 2.3, first sentence

No.	Item	Reported in
13b	Whether clinical information and index test results were available to the assessors of the reference standard	Section 2.2, paragraph 2
**METHODS — Analysis**		
14	Methods for estimating or comparing measures of diagnostic accuracy	Section 2.5
15	How indeterminate index test or reference standard results were handled	Section 2.2 (reader disagreement adjudicated); Section 3.7 (intermediate tier reported, not excluded)
16	How missing data on the index test and reference standard were handled	Not applicable — no missing data; all 250 segments had both index test and reference standard results (Section 3.1)
17	Any analyses of variability in diagnostic accuracy, distinguishing pre-specified from exploratory	Section 2.5; Section 3.4 (61-threshold sweep, exploratory); Section 3.1 and Table 2 (per-level distribution)
18	Intended sample size and how it was determined	Section 2.5, final paragraph (no a priori sample-size calculation)
**RESULTS — Participants**		
19	Flow of participants, using a diagram	Figure 3 (STARD diagram)
20	Baseline demographic and clinical characteristics of participants	Table 1; Section 3.1
21a	Distribution of severity of disease in those with the target condition	Table 1 (shift F in reference-positive segments); Table 2 (by level)
21b	Distribution of alternative diagnoses in those without the target condition	Section 2.4 and Figure 3 (retrolisthesis excluded by design); Table 4
22	Time interval and any clinical interventions between index test and reference standard	Section 2.2 and 2.3 — both read from the same radiograph; no interval and no intervention
**RESULTS — Test results**		
23	Cross tabulation of the index test results (or their distribution) by the results of the reference standard	Table 4; Table 8; Figures 4 and 7a
24	Estimates of diagnostic accuracy and their precision (such as 95% confidence intervals)	Tables 5–8; Sections 3.4–3.7
25	Any adverse events from performing the index test or the reference standard	Not applicable — both tests were read from radiographs already acquired for clinical care; no additional procedure was performed
**DISCUSSION**		
26	Study limitations, including sources of potential bias, statistical uncertainty, and generalizability	Section 4.5
27	Implications for practice, including the intended use and clinical role of the index test	Sections 4.4 and 5
**OTHER INFORMATION**		
28	Registration number and name of registry	Not registered — retrospective diagnostic accuracy analysis within the PROGRES observational cohort; registration is not required for purely observational studies (Section 2.1).

No.	Item	Reported in
29	Where the full study protocol can be accessed	The full study protocol is available from the corresponding author on request (Data Availability Statement).
30	Sources of funding and other support; role of funders	Funding statement (back matter)

Bossuyt P.M., Reitsma J.B., Bruns D.E., Gatsonis C.A., Glasziou P.P., Irwig L., Lijmer J.G., Moher D., Rennie D., de Vet H.C.W., et al. STARD 2015: an updated list of essential items for reporting diagnostic accuracy studies. BMJ 2015, 351, h5527. Checklist available at https://www.equator-network.org/reporting-guidelines/stard.
