# Supplementary material for: Quadrilateral Segmental Analysis (QSA): A Calibration-Free Geometric Method for Detecting Lumbar Spondylolisthesis
Source: Diagnostics (Basel). 2026 Oct 10;16(20):3291. doi: 10.3390/diagnostics16203291 (PMC13703141; doi:10.3390/diagnostics16203291)
Supplement: Supplementary file 1 [file diagnostics-16-03291-s001.zip › QSA_Checklist_STROBE.pdf]

## Supplementary Material

# Quadrilateral Segmental Analysis (QSA): A Calibration-Free Geometric Method for Detecting Lumbar Spondylolisthesis

*Szczepański T., Jasiński M., Koźba-Gosztyła M., Więcław J., Szymczyk P., Bladowska J., Czapiga B.*

## STROBE checklist for cohort studies

*Applies to the PROGRES cohort study, from which the data analysed here were drawn. Section numbers refer to the submitted manuscript.*

| No. | Item | Reported in |
|---|---|---|
| **TITLE AND ABSTRACT** | | |
| 1 | (a) Indicate the study's design with a commonly used term in the title or the abstract. (b) Provide in the abstract an informative and balanced summary of what was done and what was found | Title; Abstract |
| **INTRODUCTION** | | |
| 2 | Background/rationale: explain the scientific background and rationale for the investigation being reported | Section 1, paragraphs 1–4 |
| 3 | Objectives: state specific objectives, including any prespecified hypotheses | Section 1, final paragraph |
| **METHODS** | | |
| 4 | Study design: present key elements of study design early in the paper | Section 2.1, first sentence |
| 5 | Setting: describe the setting, locations, and relevant dates, including periods of recruitment, exposure, follow-up, and data collection | Section 2.1, paragraphs 1–2 (4th Military Clinical Hospital, Wrocław; May 2025–May 2026) |
| 6 | Participants: (a) give the eligibility criteria, and the sources and methods of selection of participants; describe methods of follow-up. (b) For matched studies, give matching criteria and number of exposed and unexposed | Section 2.1, paragraph 2. (b) Not applicable — unmatched design |
| 7 | Variables: clearly define all outcomes, exposures, predictors, potential confounders, and effect modifiers. Give diagnostic criteria, if applicable | Sections 2.2 and 2.3 |
| 8 | Data sources/measurement: for each variable of interest, give sources of data and details of methods of assessment (measurement). Describe comparability of assessment methods if there is more than one group | Sections 2.2 and 2.3; Section 3.2 (inter-reader agreement) |
| 9 | Bias: describe any efforts to address potential sources of bias | Sections 2.2 and 2.3 (independent blinded reading, adjudication); Section 2.5 (patient-cluster bootstrap); Section 4.5 |
| 10 | Study size: explain how the study size was arrived at | Section 2.5, final paragraph; Section 2.1 (all consecutive patients in the enrolment window) |
| 11 | Quantitative variables: explain how quantitative variables were handled in the analyses. If applicable, describe which groupings were chosen and why | Section 2.5; Section 3.7 (rounding of K to one decimal) |

| No. | Item | Reported in |
|---|---|---|
| 12 | Statistical methods: (a) describe all statistical methods, including those used to control for confounding. (b) Describe any methods used to examine subgroups and interactions. (c) Explain how missing data were addressed. (d) If applicable, explain how loss to follow-up was addressed. (e) Describe any sensitivity analyses | Section 2.5. (b) Section 3.1 and Table 2 (by level). (c) No missing data. (d) Not applicable — cross-sectional preoperative analysis. (e) Section 3.4 (threshold sweep); Section 3.6 (LOPO-CV) |
| **RESULTS** | | |
| 13 | Participants: (a) report numbers of individuals at each stage of the study. (b) Give reasons for non-participation at each stage. (c) Consider use of a flow diagram | Figure 3; Section 3.1; Table 2 |
| 14 | Descriptive data: (a) give characteristics of study participants and information on exposures and potential confounders. (b) Indicate number of participants with missing data for each variable of interest. (c) Summarise follow-up time | Table 1; Section 3.1. (b) No missing data. (c) Not applicable |
| 15 | Outcome data: report numbers of outcome events or summary measures over time | Section 3.1; Tables 1 and 2 |
| 16 | Main results: (a) give unadjusted estimates and, if applicable, confounder-adjusted estimates and their precision. (b) Report category boundaries when continuous variables were categorized. (c) If relevant, consider translating estimates of relative risk into absolute risk | Sections 3.3–3.7; Tables 4–8. (b) Tables 5 and 8. (c) Table 8 (post-test probabilities) |
| 17 | Other analyses: report other analyses done — e.g. analyses of subgroups and interactions, and sensitivity analyses | Sections 3.5 and 3.6; Tables 6 and 7 |
| **DISCUSSION** | | |
| 18 | Key results: summarise key results with reference to study objectives | Section 4.1 |
| 19 | Limitations: discuss limitations of the study, taking into account sources of potential bias or imprecision. Discuss both direction and magnitude of any potential bias | Section 4.5 |
| 20 | Interpretation: give a cautious overall interpretation of results considering objectives, limitations, multiplicity of analyses, results from similar studies, and other relevant evidence | Sections 4.2–4.4; Section 5 |
| 21 | Generalisability: discuss the generalisability (external validity) of the study results | Section 4.5, paragraph 2 |
| **OTHER INFORMATION** | | |
| 22 | Funding: give the source of funding and the role of the funders for the present study and, if applicable, for the original study on which the present article is based | Funding statement (back matter) |

*von Elm E., Altman D.G., Egger M., Pocock S.J., Gøtzsche P.C., Vandenbroucke J.P.; STROBE Initiative. The Strengthening the Reporting of Observational Studies in Epidemiology (STROBE) statement: guidelines for reporting observational studies. Lancet 2007, 370, 1453–1457. Checklist available at https://www.strobe-statement.org.*
