# Supplementary material for: Quadrilateral Segmental Analysis (QSA): A Calibration-Free Geometric Method for Detecting Lumbar Spondylolisthesis
Source: Diagnostics (Basel). 2026 Oct 10;16(20):3291. doi: 10.3390/diagnostics16203291 (PMC13703141; doi:10.3390/diagnostics16203291)
Supplement: Supplementary file 1 [file diagnostics-16-03291-s001.zip › QSA_Supplementary_File_S1.pdf]

Supplementary File S1. Reader instructions for the reference standard and endpoint-placement rules for the index test

Quadrilateral Segmental Analysis (QSA): A Calibration-Free Geometric Method for Detecting Lumbar Spondylolisthesis

A. Reference standard: instructions to the radiologists

Each radiologist classified every motion segment from L1–L2 to L5–S1 as anterolisthesis present or absent (binary verdict) by holistic visual assessment of the standing lateral radiograph. The verdict did not rest on numerical measurements alone, and no digital measurement tools were used. Translation was estimated visually, with ≥3 mm or a slip of ≥5% as alternative thresholds. The assessment was guided by eight pre-specified features (Table S1.1), three geometric and five non-geometric; there was no fixed scoring rule.

Table S1.1. Pre-specified features and their operational definitions.

ID	Feature	Operational definition	Type
a	Translation (Meyerding)	Anterior translation of the cranial vertebra, assessed along the postero-anterior axis through the posterior corners of adjacent vertebrae.	Geometric
b	Posterior vertebral body line	Line tangential to the posterior border of the vertebral body.	Geometric
c	Disc height asymmetry	Difference between anterior and posterior disc height.	Geometric
d	Vacuum disc phenomenon (Knutsson sign)	Linear or crescent-shaped lucency of gas density within the intervertebral space, not explained by overlying bowel gas.	Non-geometric
e	Disc space narrowing at the index level	Intervertebral space height at the level of the slip clearly reduced relative to the adjacent levels (Kellgren–Lawrence grade ≥3; difference in height relative to the neighbouring level).	Non-geometric
f	Traction spur (Macnab spur)	Horizontally oriented osteophyte ≥2 mm arising from the vertebral body margin 2–3 mm from the endplate and not curving towards the adjacent vertebra.	Non-geometric
g	Spinous process step-off	Discontinuity of at least one spinous-process width in the smooth arc joining the spinous-process tips on the lateral radiograph; in degenerative spondylolisthesis the step-off lies below the level of the slip.	Non-geometric
h	Baastrup sign (kissing spinous processes)	Direct contact or an interspinous gap of <2 mm between two adjacent spinous processes, with flattening or sclerosis of the opposing surfaces.	Non-geometric

The readers were blinded to the QSA output and to each other’s ratings. Segments with discordant verdicts were referred to a third, senior radiologist, blinded to the index test and to the identity of the disagreeing readers, whose verdict was final.

Supplementary File S1. Reader instructions for the reference standard and endpoint-placement rules for the index test

Quadrilateral Segmental Analysis (QSA): A Calibration-Free Geometric Method for Detecting Lumbar Spondylolisthesis

B. Index test: endpoint-placement rules

A single experienced spine surgeon, blinded to the radiologists' ratings, drew one straight line along the superior endplate of L1, L2, L3, L4, L5 and S1 (six lines per patient), following these rules:

- Each line runs along the superior endplate from its anterior to its posterior rim, excluding osteophytes.
- On concave endplates, the line joins the anterior and posterior rim points.
- Double contours are resolved by drawing the line midway between them.
- At S1, the line runs from the posterosuperior to the anterosuperior corner of the sacral endplate.

Annotated images were processed by an automated Python pipeline that detected the lines by color segmentation and fitted each line by principal component analysis. The endpoints of each line were the extreme projections of the annotated pixels on its principal axis. The QSA parameters were computed from these endpoints as described in Appendix A of the main text.
