# Supplementary material for: Quadrilateral Segmental Analysis (QSA): A Calibration-Free Geometric Method for Detecting Lumbar Spondylolisthesis
Source: Diagnostics (Basel). 2026 Oct 10;16(20):3291. doi: 10.3390/diagnostics16203291 (PMC13703141; doi:10.3390/diagnostics16203291)
Supplement: Supplementary file 1 [file diagnostics-16-03291-s001.zip › QSA_Supplementary_Table_S1.pdf]

Table S1. Fold-specific tier boundaries, N cut-offs and selected predictor sets in leave-one-patient-out validation (50 folds).

Held-out patient (study ID)	Lower K boundary	Upper K boundary	N cut-off	Selected predictor set
002	0.850	0.946	93°	K + N + |F|
005	0.850	0.946	93°	K + N + |F|
008	0.850	0.946	93°	K + N + |F|
012	0.850	0.946	93°	K + N + |F|
020	0.850	0.946	93°	K + N + |F|
023	0.850	0.946	93°	K + N + |F|
024	0.850	0.946	93°	K + N + |F|
027	0.850	0.946	93°	K + N + |F|
037	0.850	0.946	93°	K + N + |F|
044	0.850	0.946	93°	K + N + |F|
046	0.850	0.946	93°	K + N + |F|
047	0.850	0.946	93°	K + N + |F|
060	0.850	0.946	93°	K + N + |F|
065	0.850	0.946	93°	K + N + |F|
067	0.850	0.946	93°	K + N + |F|
081	0.850	0.946	93°	K + N + |F|
089	0.850	0.946	93°	K + N + |F|
090	0.850	0.946	93°	K + N + |F|
098	0.850	0.946	93°	K + N + |F|
099	0.850	0.946	93°	K + N + |F|
102	0.850	0.946	93°	K + N + |F|
103	0.850	0.946	93°	K + N + |F|
121	0.850	0.946	93°	K + N + |F|
128	0.850	0.946	93°	K + N + |F|
140	0.850	0.946	93°	K + N + |F|
141	0.850	0.946	93°	K + N + |F|
143	0.850	0.946	93°	K + N + |F|
144	0.850	0.946	93°	K + N + |F|
149	0.850	0.946	93°	K + N + |F|
157	0.850	0.946	93°	K + N + |F|
167	0.850	0.946	93°	K + N + |F|
174	0.850	0.946	93°	K + N + |F|
175	0.850	0.946	93°	K + N + |F|
176	0.850	0.946	93°	K + N + |F|
194	0.850	0.946	93°	K + N + |F|
195	0.850	0.946	93°	K + N + |F|
198	0.850	0.946	93°	K + N + |F|
199	0.869	0.946	93°	K + N + |F|
202	0.850	0.946	93°	K + N + |F|

Held-out patient (study ID)	Lower K boundary	Upper K boundary	N cut-off	Selected predictor set
203	0.850	0.946	93°	K + N + |F|
216	0.850	0.946	93°	K + N + |F|
217	0.850	0.946	93°	K + N + |F|
219	0.850	0.946	93°	K + N + |F|
221	0.850	0.946	93°	K + N + |F|
223	0.850	0.946	93°	K + N + |F|
225	0.850	0.946	93°	K + N + |F|
230	0.850	0.946	93°	K + N + |F|
231	0.850	0.946	93°	K + N + |F|
232	0.850	0.946	93°	K + N + |F|
235	0.850	0.943	93°	K + N + |F|

In each fold, all data-dependent steps used the 49 training patients only. Lower K boundary: lowest K among training reference-negative segments (held-out K below it: rule-in tier). Upper K boundary: highest K among training reference-positive segments (held-out K above it: rule-out tier); K equal to a boundary was assigned to the intermediate tier, without rounding. N cut-off: Youden optimum among the observed N values of the training set (test positive if N ≤ cut-off), ties resolved to the lowest cut-off. Selected predictor set: candidate model with the highest inner leave-one-patient-out AUC within the training set, ties resolved to the earlier model in the pre-specified order (Table 7).
